# Artificial Vichy Water

**Published:** 1851-08

**Authors:** 


*Artificial Vichy Water.*—Bicarbonate of soda, dissolved in water, imitates the Vichy water very imperfectly. The *Bulletin de Therapeutique* gives the following formula for the purpose:—Bicarbonate of soda, 75 grains; chloride of sodium, 4 grains; sulphate of soda, 10 grains; sulphate of magnesia, 3 grains; sulphate of iron, one-fifth of a grain. Dissolve in a bottle of water, and use it as the natural Vichy Water. By adding 45 grains of citric acid, the water will effervesce.—*Ibid.*

